# Prediction of Antioxidant Activity of Cherry Fruits from UAS Multispectral Imagery Using Machine Learning

**DOI:** 10.3390/antiox9020156

**Published:** 2020-02-14

**Authors:** Christos Karydas, Miltiadis Iatrou, Dimitrios Kouretas, Anastasia Patouna, George Iatrou, Nikolaos Lazos, Sandra Gewehr, Xanthi Tseni, Fotis Tekos, Zois Zartaloudis, Evangelos Mainos, Spiros Mourelatos

**Affiliations:** 1 Ecodevelopment S.A., Environmental Applications, 57010 Thessaloniki, Greece; iatrou@ecodev.gr (G.I.); lazos@ecodev.gr (N.L.); gewehr@ecodev.gr (S.G.); tseni@ecodev.gr (X.T.); smourelat@gmail.com (S.M.); 2 Agroecosystem L.P., Research and Trade of Agricultural Products, 63200 Nea Moudania, Greece; m_iatrou@ecodev.gr (M.I.); zoiszartaloudis@gmail.com (Z.Z.); 3 Laboratory of Animal Physiology, Dept. of Biochemistry and Biotechnology, University of Thessaly, 41500 Larissa, Greece; dkouret@gmail.com (D.K.); anastasia.pat93@hotmail.com (A.P.); fotis.tekos@gmail.com (F.T.); 4 Novagreen S.A., Agricultural Supplies, 58001 Giannitsa, Greece; mainos_e@hotmail.com

**Keywords:** antioxidant activity, machine learning, drones, precision farming

## Abstract

In this research, a model for the estimation of antioxidant content in cherry fruits from multispectral imagery acquired from drones was developed, based on machine learning methods. For two consecutive cultivation years, the trees were sampled on different dates and then analysed for their fruits’ radical scavenging activity (DPPH) and Folin–Ciocalteu (FCR) reducing capacity. Multispectral images from unmanned aerial vehicles were acquired on the same dates with fruit sampling. Soil samples were collected throughout the study fields at the end of the season. Topographic, hydrographic and weather data also were included in modelling. First-year data were used for model-fitting, whereas second-year data for testing. Spatial autocorrelation tests indicated unbiased sampling and, moreover, allowed restriction of modelling input parameters to a smaller group. The optimum model employs 24 input variables resulting in a 6.74 root mean square error. Provided that soil profiles and other ancillary data are known in advance of the cultivation season, capturing drone images in critical growth phases, together with contemporary weather data, can support site- and time-specific harvesting. It could also support site-specific treatments (precision farming) for improving fruit quality in the long-term, with analogous marketing perspectives.

## 1. Introduction

According to Food and Agriculture Organization (FAO) data, the agricultural land cultivated with cherries exceeded 0.4 million hectares over the last decade (2007–2017), with slight upward trends, whereas world production reached 2.5 million tonnes. Greece ranks 12th in cherry production worldwide, with 100 thousand hectares, of which ~75% is in the Region of Central Macedonia, mainly in Pella and Imathia regional units [1]. Cherry cultivars of Greece belong to the species of *Prunus avium*, family Rosaceae. Cherry trees need a certain number of cold nights to break dormancy and bloom, and they prefer soils with pH levels between 6.0 and 6.8. Most varieties require cross-pollination [2].

Cherry fruits are rich in polyphenols, mainly flavonoids, flavanols and flavonols [3,4]. Interestingly, the colour of the crops depends on anthocyanin levels, a flavonoid compound, during maturation stage [5]. Polyphenols are secondary metabolites of plants that contribute to bitterness, colour, odour and protection against UV radiation and pathogens [6]. Important constituents of human diet such as coffee, pomegranate, olive oil, grapes/wine, different kinds of fruits and vegetables possess high nutritional value because they are rich in polyphenolic compounds [7,8]. One of the most remarkable properties of polyphenols is their ability to act as antioxidants in vivo. Specifically, numerous studies have reported that polyphenolic compounds exert anti-inflammatory [9,10], cardioprotective [11], cancer chemopreventive [10,12,13] and neuroprotective properties [14,15] because of their antioxidant content [16].

Apart from polyphenols, cherries are also a good source of carotenoids, vitamin C and potassium [17], tryptophan, serotonin and melatonin [18,19], which also contribute to the strong antioxidant activity of cherries [20]. Moreover, they contain remarkable amounts of sugars whose bioaccumulation depends on the cultivar, several agronomic factors, the environmental conditions and the stage of maturation [21]. Sugars are necessary for plant survival as they depict the energy state of the crops and, thus, the ability of cherries to manage their energy supplies [22]. Different types of sugars, such as glucose, sucrose, fructose, maltose and sorbitol are found in cherries. However, the main sugars are glucose and fructose representing approximately the 90% of total sugar content in cherry fruits [21].

However, the chemical composition (i.e., the amounts of polyphenols present in the fruit) of cherries depends on the cultivation practices, the environmental status and the maturation state [5,23]. Therefore, the heterogeneity within each cherry plantation, both inherent (due to soil, variety, etc.) or acquired (due to agricultural management), is expected to create a similar (albeit more complex) variation to its spatial distribution of the antioxidant content of fruits. If this variation is mapped, it will allow indirect estimation of the antioxidant content of the cherries without time-consuming laboratory measurements, and immediate identification of ready-to-harvest trees.

Remote sensing is an appropriate method for monitoring crops. In particular, the use of multispectral images with simultaneous collection plant samples offers the possibility of detecting and interpreting the geospatial variability of the physical and chemical properties of vegetation and soils. Remote sensing methods have been extensively applied to annual crops [24,25,26,27], but also to citrus fruits [28,29], avocados [27]), mangoes [26], vines [30,31,32], etc. Especially for cherries, the literature is limited: most studies [33] have focused on the mapping techniques of cherry trees. Based on object-oriented image analysis on unmanned aerial systems (UAS) data, Karydas et al. [34] developed a method for detailed mapping of tree crown in olive plantations. Earlier, Iatrou et al. [35] had used multispectral images acquired also with UAS for detecting verticillium disease in olive trees.

Generally, there is a lack of knowledge for the relationship between polyphenols content, soil properties and agricultural practices [36]. When plants are grown in resource-rich environments, growth is promoted over the production of secondary metabolism, and thus the production of secondary metabolites, such as polyphenols, is reduced. As Delgado et al. [37] point out, nitrogen fertilization decreases polyphenol content in most cases. According to Oliveira et al. [23], phosphorus deficiency promotes anthocyanin content, while potassium at levels above the optimum improved the contents of phenolic compounds. Finally, Zhou et al. [38] showed that sulphur addition promoted total phenolics and decreased anthocyanins contents. 

Useful information with regards to polyphenol content changes can be acquired by the reflectance of sun irradiance of leaves and canopies based on changes of leaf carotenoid and polyphenol content [39]. Carotenoid and polyphenol variations are observed due to environmental stress to prevent oxidative damage in the photosystem [40,41,42]. Chlorophyll changes are also indicative of environmental stress, as chlorophyll is involved in the process of photosynthesis [43]. The growing recognition of the health benefits of cherry fruits makes challenging the detection of cherry trees bearing fruits with the highest content of antioxidants using remote sensing techniques. 

The aim of the current work is to study the relationship between the remote sensing indices (such as carotenoid, polyphenol and chlorophyll indices) and the antioxidant activity of cherries, along with the soil attributes and develop a prediction model for the detection of the antioxidant content in cherry fruits from remote sensing imagery in near real-time. The basic hypothesis of the research was that remote sensing images from UAS in combination with soil data are necessary and efficient in detecting antioxidant content in cherries in advance of harvesting. The operational perspective of this research is to propose site- and time-specific harvesting of cherry plantations based on soil and image data in almost real time.

## 2. Materials and Methods 

### 2.1. Study Area

**Figure 1:**
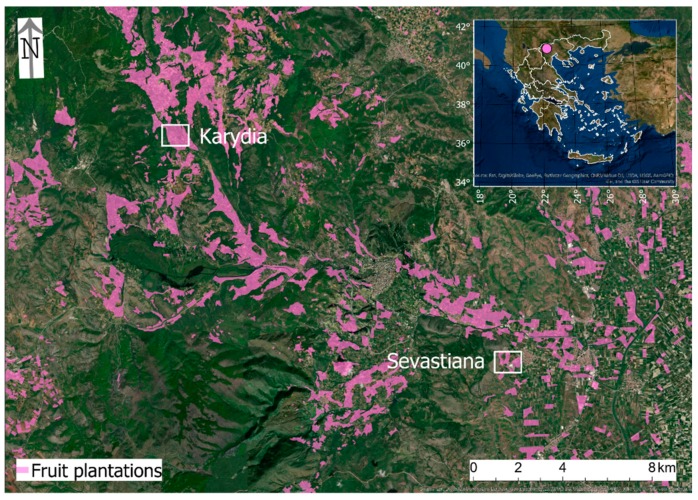
The location of the study area and the two study sites of the cherry plantations.

The study area is situated in the regional unit of Pella, in the northern part of Greece, known for fruit production, such as peaches, apricots, apples, cherries, etc. Two sites with different environmental characteristics were targeted, as representative of the whole study area (Figure 1).

The first site is located close to Sevastiana village, 7 km SE of the city of Edessa, between 70 and 100 m height, at the foot of mountain Vermio. The four studied fields cover a total area of ~1.87 ha, with the cherry varieties of Lapins, Sabrina, Early Lory, Canada Giant and Sweet Early, which are characterized as early cherry varieties. In most of the fields, two–six cherry varieties coexisted. The cultivation scheme varied between cup-shaped with planting distances 4 × 4.5 m or 4.5 × 4.5 m and palmettes with planting distances 3.5 × 2.5 or 3.5 × 1.5 m.

The second study site is located at Karydia region, 9 km NW of the city of Edessa, between 750 and 850 m height, at the foot of mountain Vorras. The mountainous landscape in this area has resulted in irregular field shapes. The studied fields covered a total area of 1.54 ha, with the cherry varieties of Ferrovia, Hedelfinger, Germersdorfer and Bakirtzeika, following late maturity. Again, in most of the fields, more than one cherry variety coexisted. The cultivation scheme in all cases was cup-shaped, with planting distances from 4.5 × 4.5 m to 6 × 6.5 m depending on the variety, the location and the shape of the field.

### 2.2. Experimental Design

The development of the prediction model was based on machine learning methods applied on data collected from the study area in two consecutive cultivation seasons (2018 and 2019). During the first year, fruits and leaves from 238 trees were sampled on nine different dates and then analysed for their antioxidant content and taste (sugar content). In parallel with the fruit and leaves sample collection, a set of multispectral and RGB images were captured from both study sites. These data were used for model building, whereas another 49 trees were imaged, sampled and analysed in the second year for validation purposes. 

After the end of both cultivation seasons (in October of 2018 and 2019, respectively), soil sampling field surveys were conducted to get the required information about soil. In 2018, a set of 64 soil samples were collected, from which soil property surfaces were created with interpolation; while, in 2019, the soil dataset comprised 19 samples (repeated sampling of about one third of previous locations) (Table 1).

**Figure 2:**
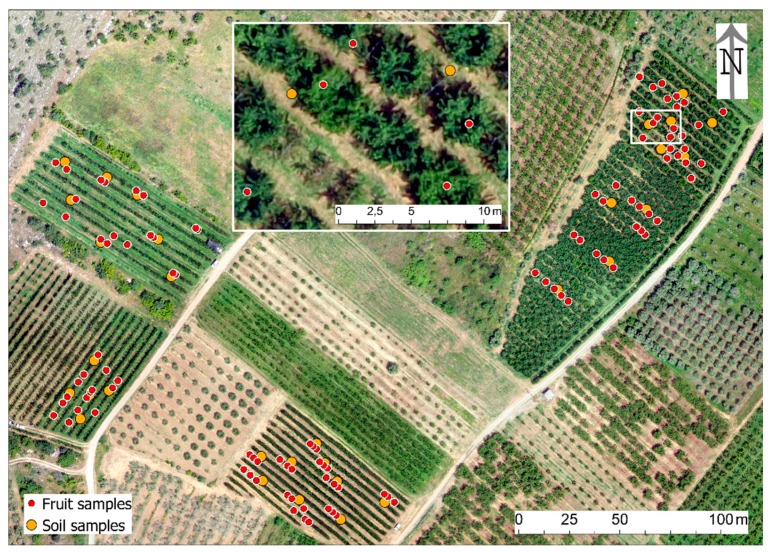
The fruit and soil samples in Sevastiana (a close view in the inset).

**Figure 3:**
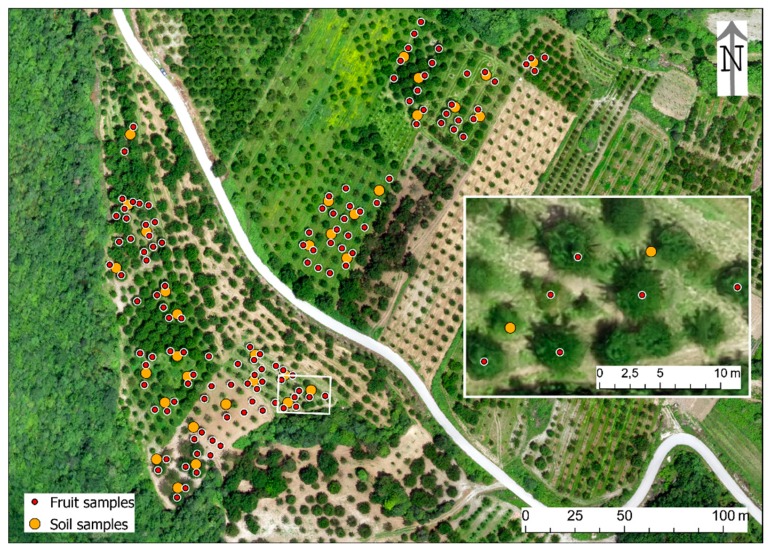
The fruit and soil samples in Karydia (a close view in the inset).

The sampling points were preceded by an autopsy in the study sites and the logging of the trees one by one in order to create a tree register, where the location of all the trees corresponded to a grid (planting line × planting point), with their respective variety. The existence of more than one variety per parcel, intermediate plantings (mainly pollinators) and the existence of empty spaces between the trees made this procedure mandatory to avoid allocation errors. The first Red Green Blue (RGB) image from UAS for each of the study sites was then taken as background for further mapping in a Geographic Information System (GIS) (Figure 2 and Figure 3).

### 2.3. Data Set Preparation

#### 2.3.1. Image Data

The equipment for imaging the study area consisted of an unmanned aerial system (UAS); namely, e-Bee; three digital cameras; and flight design and image processing software, respectively. The e-Bee vehicle was a fixed-wing plane, specialized for agricultural use. It can carry a variety of cartographic cameras in the visual or infrared wavelengths. The cameras employed are as follows.

- A modified ‘multiSPEC 4C’ camera, with four lenses, capturing at the following wavelengths; 510 nm, 660 nm, 710 nm and 790 nm (modification concerned changing wavelength of the first band to 510 nm from 550 and wavelength of the third band to 710 nm from 735 nm).
- A ‘Parrot SEQUOIA’ camera (Parrot Drones SA, Paris, France), with five lenses, capturing at the following wavelengths; 550 nm, 660 nm, 735 nm and 790 nm.
- A Canon S 110 camera, with a single lens, capturing RGB images.

The flights were designed using eMotion software, at an altitude of 115 m on average, resulting in a ground sampling distance (pixel) of 12 cm for the multiSPEC 4C and Parrot SEQUOIA images and 4.1 cm for the Canon S110 images. All the images were acquired before midday for optimum imaging results. In advance of image acquisition, ground control points (GCPs) were placed appropriately using differential GPS and test flights were executed in order to estimate the flight needs for each of the study sites. All the images were preprocessed with Pix4dmapper software, to produce a set of excellent quality orthomosaics for each of the study sites.

In the first experimental year (2018), nine flights were conducted, covering a period of 52 days in total, with intervals varying from 2 to 8 days, just before (Karydia) or during (Sevastiana) fruit harvesting. In the second experimental year (2019), five new flights were conducted using the same cameras and flying parameters as in the first year. Two of the flights were executed at Sevastiana, just before and during harvesting period, whereas the remaining three ones were executed at Karydia before harvesting. The flights covered a period of 42 days in total, with intervals varying from 10 to 12 days. 

The broad spectral resolution of UAS images enabled the computation of vegetation indices which according to the literature, are significantly correlated with various vegetative properties of many cultivated species [44]. Three indices were computed in this research: the Normalized Difference Vegetation Index (NDVI), as an expression of plant biomass and vigor; the Carotenoid Reflectance Index 2 (CRI2), as an expression of carotenoid content in the leaves [45]; and the Anthocyanin Reflectance Index (ARI), as an expression of polyphenol content in the leaves [46]. The equations used for the computation of the above spectral indices were

NDVI = (R790 − R660)/(R790 + R660)(1)

CRI2 = [(1/R510) − (1/R710)] × R790(2)

ARI = (1/R550) − (1/R790)(3)

where Rnnn is the reflectance recorded by the two multispectral cameras used at nnn wavelength (nm). These wavelengths were selected among the available by the two cameras, as being closer to the required wavelengths defined in the literature; for example, for ARI index (see Equation (3)), reflectance values at 790 nm, which are supplied either by multiSPEC 4C or SEQUOIA camera, are found closer to the reflectance values at 760 nm in the spectral signature of vegetation, compared to other available wavelengths; 760 nm is the exact wavelength required by the original ARI equation. It is worth mentioning that computation of CRI2 would not be possible without the modification of the original multiSPEC 4C camera.

#### 2.3.2. Fruit Samples

The collection of cherry fruit and leaf samples took part from 28 April to 13 June 2018 and from 10 May to 21 June 2019. Every sampled tree was harvested only once to avoid fruit sample disturbance in case of harvesting the same tree for several consequent dates.

Each fruit sample consisted of 50 individual fruits collected from different tree sections (heights and sides), only from the annual shoots, and similarly, the leaf samples. All the possible varieties found in the examined fields were sampled at different dates from both sites and for both experimental years (Table 2 and Table 3). The samples were transported to the laboratory in boxes full of ice placed carefully in air-tight closed bags. The samples were kept at −20 °C until the analyses were conducted.

Seven fruits from each sample weighing 2 g in total were randomly chosen and slashed into small pieces. The homogenization was conducted using distilled water (dH_2_O) at a portion of 1:2 (*w*/*v*) (homogenizer IKA^®^ ULTRA-TURRAX^®^ T18, IKA^®^-Werke GmbH & Co. KG, Staufen, Germany) followed by sonication (70 amplitude, 0.5 cycle, 1 min, ice). The homogenates were then centrifuged (10,000× *g*, 15 min, 4 °C), and the supernatant was collected and stored at −80 °C for further analysis.

The total reducing capacity of the samples was determined using the Folin–Ciocalteu (FCR) reagent [47]. The reaction includes dH_2_O (1 mL), the Folin–Ciocalteu reagent (100 μL) and 20 μL of each sample, and the mixture was incubated for 3 min in the dark. Afterwards, 25% w/v solution of sodium carbonate (280 μL) and dH_2_O (600 μL) were added, and the samples were incubated for 1 h at RT in the dark and the absorbance was monitored at 765 nm. As the blank a sample containing the Folin–Ciocalteu reagent and dH_2_O was used. The Folin−Ciocalteu reducing capacity was determined using a standard curve of gallic acid (0, 50, 150, 250, 500 μg/mL) and is expressed as mg of gallic acid per gr of each sample [48]. 

For the 2,2-diphenyl-1-picrylhydrazyl (DPPH•)• radical assay determination, methanol, DPPH• (100 μM) and a wide range of cherry fruit concentrations (the cherry fruit concentrations studied are 1.56, 3.12, 6.25, 12.5 and 25 mg/mL) in a total volume of 1 mL were mixed and incubated at room temperature (RT) for 20 min in the dark. Then, the optical density was monitored at 517 nm. The results were expressed as the IC50 value, which indicates the cherry extract concentration that induced 50% scavenging of the DPPH radical. In each experiment, the tested sample alone in methanol was used as blank and DPPH• alone in methanol was used as control [48]. The 2,2-Azino-bis-(3-ethylbenzothiazoline-6-sulfonic acid) (ABTS•^+^) radical scavenging capacity of the samples was determined according to Cano et al. [49] with slight modifications [48]. In brief, dH_2_O, ABTS (1 mM), H_2_O_2_ (30 mΜ) and horse radish peroxidase (HRP) (6 mΜ) were mixed in a total volume of 1 mL and incubated at RT for 45 min in the dark. Then, 50 μL of different concentrations of the samples were added and the optical density was monitored in 730 nm. In each experiment, a sample without HRP was used as the negative control [48]. The results were expressed as the IC50 value, which indicates the cherry fruit concentration that induced 50% scavenging of the ABTS radical.

Total soluble solids were used as an estimate of sugar content of fruit, because sugars are the major soluble % solid in fruit. The levels of the sugars were evaluated with a hand-held digital pocket refractometer (Atago Co., PAL-1, Tokyo, Japan). For the measurement, 200 μL of cherry juice was squeezed from a single cherry fruit, it was purred in the refractometer sensor and the value was recorded after 5 s. The sugar content for five cherry fruits was assessed from each tree [50].

The leaf samples were analysed for total nitrogen, phosphorus, potassium, calcium, magnesium, boron, manganese, zinc, iron and copper. These results were useful in estimating the nutritional status of the trees, in association with soil analysis results.

#### 2.3.3. Soil Samples

In October of 2018, a field survey was conducted for soil sampling. Sixty-four (64) samples were taken from both sites (31 in Sevastiana and another 33 in Karydia), following a purposive systematic sampling scheme, with soil samples extracted from inside different clusters of trees. The collected soil samples were well distributed throughout the fields and dense enough (~1 sample every 0.6 ha on average). Similarly, in October of 2019, a second set of soil samples was collected from the same locations of 19 samples (out of 64), representative of the original set; 10 samples were collected in Sevastiana and nine in Karydia. Thus, 83 soil samples were collected in total for both cultivation years.

Every single sample was composed by extracts taken from three locations within a radius of 3 m around the sample’s GPS spot and from a depth of up to 40 cm. The collected samples were packed, coded and send for full physical and chemical analysis.

Finally, raster surfaces for all measured soil properties (16 features in total) were created with spatial interpolation of sample points using the Inverse Distance Weighting (IDW) method [51]. Testing the accuracy of the spatial interpolation outputs was out of the scope of this research. Instead, their accuracy can be implied by two facts: (a) the density of soil sampling in relation to tree sampling was very high and uniform throughout the study fields, specifically, in Sevastiana: 31 soil samples for 108 trees, or 1 soil sample per 3.48 trees, and in Karydia: 33 soil samples for 126 trees, or 1 soil sample per 3.81 trees, and (b) the spatial autocorrelation of the soil samples (tested with the Global Moran’s I index) for all measured properties revealed highly clustered samples, which implies very smooth transition from one sample to another and thus very close values between known and predicted locations.

#### 2.3.4. Terrain and Climatic Data

A digital elevation model (Digital Elevation Model, DEM) of 12.5 m spatial resolution, derived from ready-to-use ALOS PALSAR satellite data, was introduced in the geodatabase. From this dataset, the altitude (in meters), the slopes (in percent), the aspect (in azimuthal angle) and the surface water flow accumulation (in number of upstream cells) were calculated using appropriate geospatial functions.

A times series of climatic data were collected for the two study sites from the closest weather stations (Karyotissa station and Kerasia station, for Sevastiana and Karydia sites, respectively), belonging to the Greek weather station network (https://www.meteo.gr/); the following data were included; mean, maximum, and minimum temperature, rain volume, and wind speed and direction, at daily step.

### 2.4. Machine Learning

As ABTS has similar activity to DPPH, analysis was limited to DPPH and FCR. Correlations among the radical scavenging activity (DPPH) and FCR, with the physical and chemical attributes of soils, remote sensing indices, climatic, topographic and hydrographic data, were calculated using the corr() function on the Pandas DataFrame (5% probability) [52]. Principal component analysis (PCA) was also applied in this study to identify the factors influencing the antioxidant activity of the cherry fruits. PCA analysis was conducted using the R statistical software and the dimdesc function of FactorMineR package in R [53,54]. The factoextra package in R was used to identify the top contributing variables on the first principal component [55]. Differences in temperature between Sevastiana and Karydia experimental areas were analysed using the ANOVA procedure of the Genstat statistical package (VSN International, Oxford, UK) at a probability level of 0.05.

Four machine learning algorithms were tested: Extreme Gradient Boosting (XGBoost), Random Forest (RF), Support Vector Regression (SVR) and Multiple Perceptron (MLP) for the data gathered in 2018. The algorithms were parameterized for 2018 data and validated for 2019 data. Prior to modelling steps, the dataset was checked using multicollinearity diagnostics with the Mctest package in R [56]. This was aiming to detect collinearity in the dataset among regressors on the DPPH variable [56]. All variables remained after multicollinearity were centralized (centralized variable = x-mean(x))/std.dev. (x), where x is the original value) for feeding the multiple perceptron algorithm. The variables were also scaled for the support vector regression algorithm (scaled = (x-mean(x)/std.dev. (x)). Data were not transformed for the XGBoost and random forests algorithms. Then, the data from 2018 experimental season were randomly separated into training and test datasets consisting of 70% (161) and 30% (69) of the total data, respectively. The algorithms were configured in Python [57]. 

The XGBoost algorithm is a dynamic machine learning technique, as new models are created that are added to the initial to correct the prediction error. The advantage of this technique is that it filters the data, leaving out the data that fit in the weak models and focuses on the development of new models that can better deal with the remaining data [58]. Due to this technique XGBoost can perfectly model difficult problems with multiple variables. Ultimately, the aim of XGBoost is to convert a low statistical hypothesis to a more robust and stronger statistical hypothesis providing a predictive model with high prediction accuracy by combining moderately inaccurate models [59]. However, the efficiency of this algorithm relies much on the optimal selection of the hyperparameters. The XGBoost model was performed with the following optimized hyperparameters; colsample_bytree = 0.4, gamma = 0.1, learning_rate = 0.07, max_depth = 5, min_child_weight = 1.5, n_estimators = 10,000, reg_alpha = 0.00001, reg lambda = 0.45, subsample = 0.95.

Random forest is also a dynamic technique because is an improvement of bagged decision trees. The decision trees are constructed in a way that reduces the correlation between the individual trees. This is done by choosing a random sampling of training data when building trees and a random subset of features in each split. Finally, the predictions are made by averaging the predictions of each decision tree [60]. This method aids in dealing with the overfitting problem encountered by simple decision trees algorithms. The Scikit-Learn library was used for configuring the random forest algorithm [61]. The Random Forest was configured with the following parameters: max_depth = 110, min_samples_leaf = 2, min_samples_split = 2, n_estimators = 300.

SVR developed the recent years, and it has shown good performance for regression problems (SVR) [62]. The SVR is a dynamic technique that creates a hyperplane separating the data classes by maximizing the margin between the classes’ closest points. There are also two other lines, called boundary lines, which create a margin. This boundary margin separates the data classes in SVR. The main benefit of SVR compared to simple regression is that SVR tries to fit the error within a certain threshold. Thus, for example, simple regression tries to minimize the distance between the observation point and the model line, whereas SVR finds the hyperplane that has the maximum observation points within the boundary lines [63]. The Scikit-Learn library was used for configuring the SVR algorithm [61]. The selection of the hyperparameters for SVR was C = 1.0, cache_size = 200, degree = 3, epsilon = 0.1, kernel = ‘rbf’, max_iter = −1, tol = 0.001.

The MLP is a type of artificial neural network algorithm. The power of MLP, and neural networks in general, is due to their ability to learn any mathematic function between the data and the output variable. This ability is due to their hierarchical structure of these algorithms, which is based on their multilayer structure. Thus, these algorithms can model difficult problems, with nonlinear correlation between the variables, and mostly data having hierarchical structure. A network consists of layers of parallel processing elements and each layer is connected to the preceding layer by nodes having variable weights. A multilayered network consists of an input layer including the initial data, one or more hidden layers, which is an intermediate layer between the input and the output layer and the output layer, which produces the result for the given inputs [64]. The tensorflow interface was used for executing the MLP algorithm [65]. The MLP model was performed with two hidden layers having 64 nodes each. The rectified linear unit (ReLU) was utilized as activation function for both hidden layers.

## 3. Results

### 3.1. Geodatabase

A single geodatabase was constructed for the entire study area and both cultivation seasons, in order to proceed with a common analytical view for the entire data set. The cherry plantations were digitized as polygon features, whereas the cherry trees were digitized as points fitting exactly to the centre of the trees as they were recognised in the RGB images; the soil samples were digitized as points, too. From each retrieved or computed raster (e.g., NDVI, slope, etc.), the values corresponding exactly to the geometric centre of the tree were transferred to the vector tree level, using a bilinear interpolation method. Thus, a total of 34 parameters (five fruit, 16 soil, three spectral, six climatic, three topographic and one hydrographic) were assigned to the tree points.

Before the implementation of the machine learning methods, the whole dataset underwent geostatistical analysis, which in turn assisted variable set optimization. Specifically, the spatial autocorrelation test (using the Global Moran’s I index) revealed that when the samples of fruit content data set were examined by date, most variables were found randomly distributed in space. Examination by date was necessary, as date by itself was introduced in the analysis as an independent variable. On the opposite, soil and topographic–hydrographic parameters were found to be clustered, which was expected and can be attributed to the dense sampling scheme, followed in order to avoid fruit sample disturbance, which could occur if sampling the same tree for all dates (as explained earlier). Existence of randomization in the samples’ spatial distribution indicates an unbiased sampling scheme and, thus, unbiased statistical analysis.

### 3.2. Modelling

To examine the relationship between the free radical scavenging activity (DPPH), remote sensing indices, soil attributes, climatic, topographic and hydrographic data of 2018 experimental season, unsupervised machine learning analysis (the dataset has no target variable) based on PCA was performed. PCA aids in defining which of the above-mentioned parameters are more informative for the antioxidant activity of cherry fruits. Unlike the standard correlation analysis, the PCA simplifies the structure of a set of variables by replacing them with few uncorrelated linear combinations of the initial set of variables [66]. The scope of this is to describe correlations between the variables reducing the dimensionality of the original dataset. Thus, this can make the correlation structure more easily understandable and interpretable from an agronomic perspective. As the climatic data have high spatial autocorrelation, due to the location of the two experimental areas, only soil analysis, remote sensing, topographic, fruit quality and soil hydrographic data, along with the date of fruit sampling and cultivar, were included in the PCA. This judgment was made to avoid overemphasizing the contribution of the climatic data in the analysis and misinterpreting the role of the soil attributes on fruit quality. However, later, during the construction of the predictive models, multicollinearity diagnostic test was conducted for assessing the collinearity of all the variables including the climatic data.

**Figure 4:**
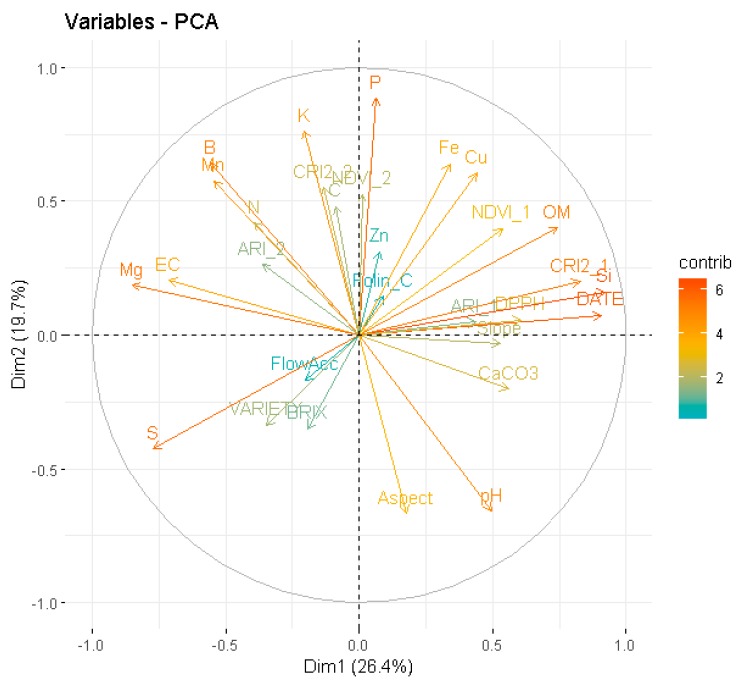
Principal component analysis (PCA) for the soil, fruit quality, remote sensing and topographic data affecting the cherry fruit free radical scavenging activity (DPPH).

**Figure 5:**
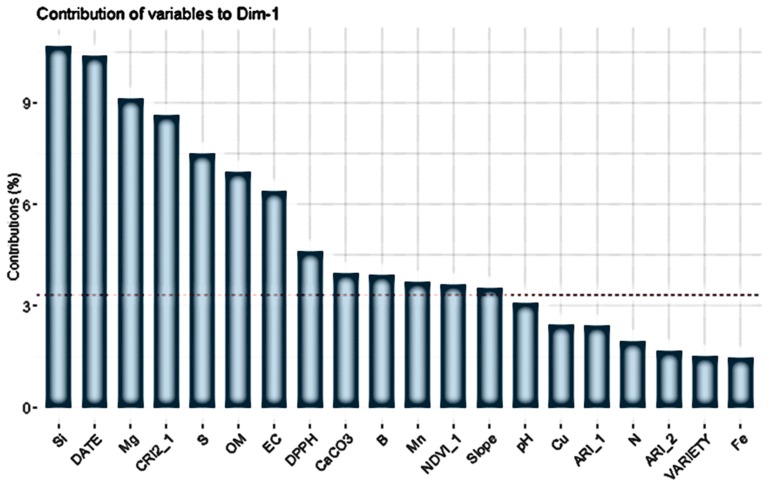
Variable contribution plot showing the 20 top variables contributing to the first principal component. The dashed line on the graph indicates that a variable with contribution larger than this cut-off could be considered as important contributing variable to the component, as determined using the dimdesc function in R.

PCA revealed that high IC_50_ values in the DPPH assay (i.e., low antioxidant activity of fruits) are on the right side, which is represented by cherry trees having high NDVI and CRI2 levels and grown on soils having high Organic Matter (OM) content (Figure 4). The first principal component (PC1) presents positive high correlation in descending order with Si, Date, CRI2_1, OM, DPPH, CaCO_3_, NDVI_1 and slope, and negative high correlation in descending order with Mg, S, EC, B and Mn, as shown in the variable contribution plot (Figure 5).

**Figure 6:**
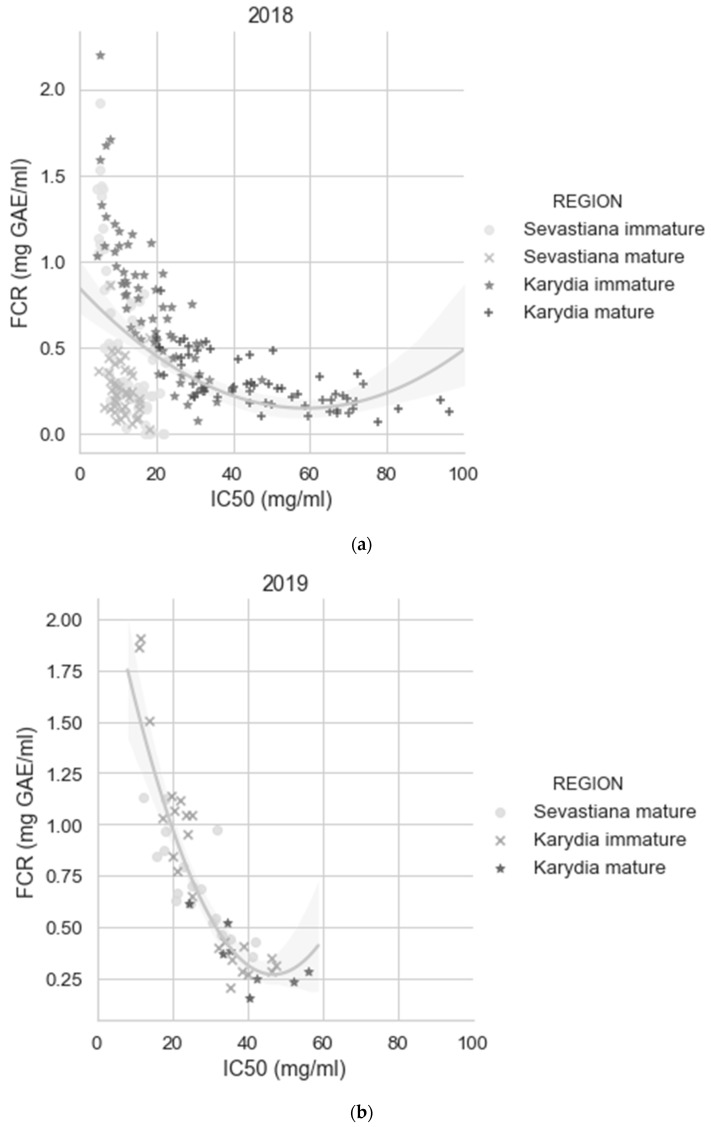
Relationships between DPPH and FCR for (**a**) 2018 (*R*^2^ = 0.21) and (**b**) 2019 (*R*^2^ = 0.81). Both graphs show that mature cherry fruits harvested at Karydia had higher than 20 mg/mL IC50 values for both 2018 and 2019.

To further explore interactions between the various variables affecting fruit quality, Pearson correlation was used, and Pearson’s coefficients are listed in Table 4. Some interesting correlations was the negative correlation of DPPH with FCR, which was an expected finding, as it is known that polyphenols correlate with the antioxidant capacity of fruits and vegetables [67,68]. Leaf analysis data were not included in the PCA because represented a small fraction of the dataset, but some of them gave interesting correlations with DPPH and FCR. The most interesting correlations were the negative correlation of leaf boron content (L_B) with DPPH and the correlation of leaf potassium content (L_K) with the FCR. Interestingly, FCR did not appear as a highly correlated variable in PC1 (Figure 5). Figure 6 shows that fitting a polynomial line gave a weak correlation between DPPH and FCR in 2018 compared to 2019, when the correlation was very strong. However, for both seasons, the IC50 values of the cherry fruits from Sevastiana experimental area in the DPPH were found below 30 mg/mL. (Figure 6). 

**Figure 7:**
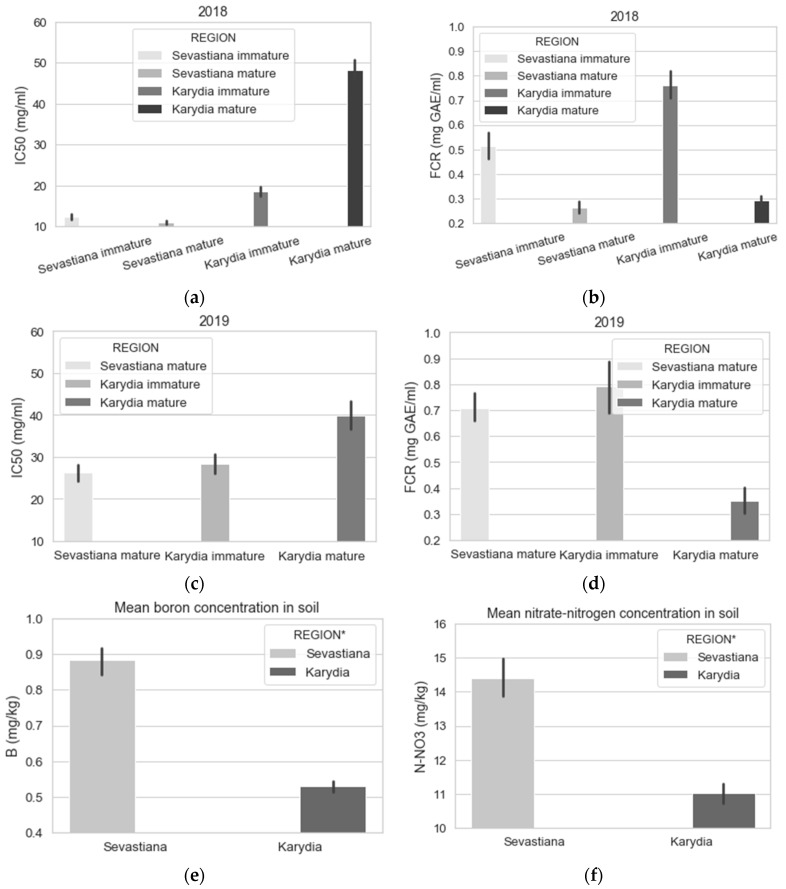
Mean IC50 values in the DPPH assay and total phenolic content (FCR) of immature and mature fruits for Sevastiana and Karydia in 2018 and 2019 (**a**–**d**). Mean soil boron and nitrate/nitrogen concentration for Sevastiana and Karydia (**e**,**f**).

Figure 7 presents the results of the fruit free radical scavenging activity (DPPH) and FCR from the two experimental areas for immature and mature harvested fruits. Figure 7e,f shows that soils in Sevastiana have significantly higher boron and nitrate/nitrogen levels compared to Karydia, because of more intensive fertilization by the growers. Fertilization for Sevastiana cherry trees is as follows; 1 kg per tree of 20−5−10+0.5B in January, 1 kg per tree of 18−9−18+0.2Fe+0.2Zn in mid-April and 1 kg per tree of 15−15−15 in mid-June. Fertilization for Karydia cherry trees is as follows; 1.5 kg per tree for trees up to 20 years old of 0−12−24+0.5Fe+0.3Zn+0.2B in mid-November and 1 kg per tree for trees up to 20 years old of 25.5−0−0+0.18Fe+0.5Zn in mid-March; for trees older than 20 years old the dose is a little higher.

**Figure 8:**
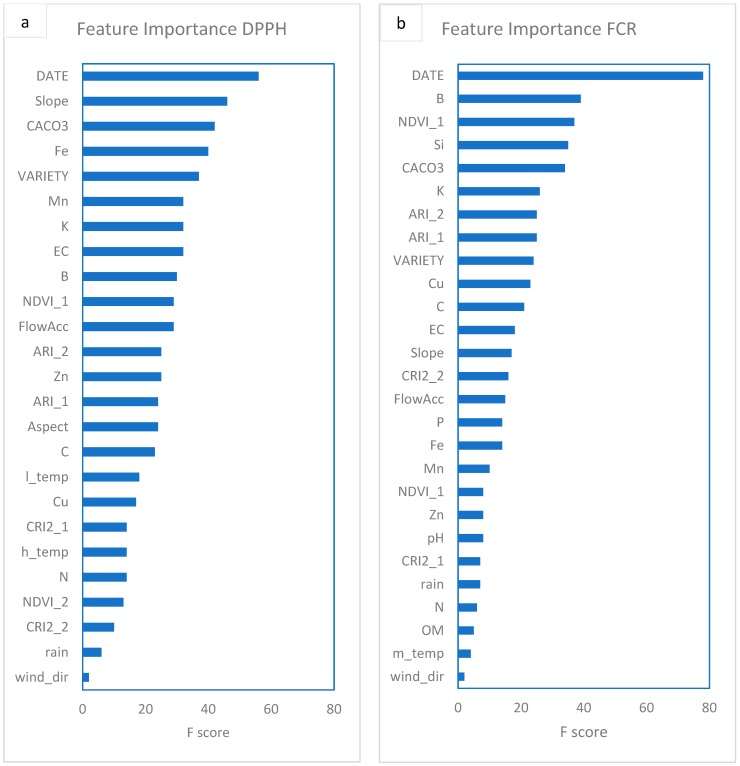
The results of feature evaluation using F-score method for DPPH (**a**) and FCR (**b**) features for 2018 data.

The XGBoost variables importance for the remote sensing indices, soil attributes, climatic, topographic and hydrographic data of 2018 experimental season for the DPPH and FCR XGBoost models is presented in Figure 8. The FCRXGBoost model was trained just for the interpretation of variables effect on FCR, whereas the DPPH XGBoost model was trained for constructing a predictive model for the detection of cherry fruits having high antioxidant capacity. In general, from the remote sensing indices, the NDVI from the first UAS flight was the most important remote sensing index for both models followed by ARI. Interestingly a topographic variable (slope) was considered as the second most important variable from the XGBoost model with regards to the DPPH prediction. From Figure 8, it is evident that boron is equally important for both DPPH and FCR models.

**Figure 9:**
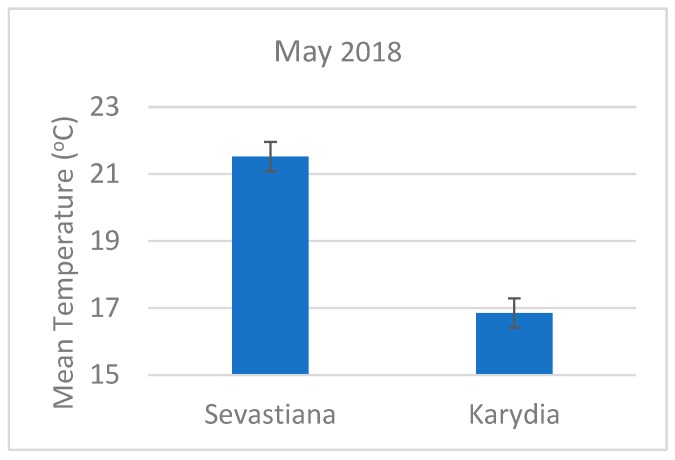
Mean May temperature for Sevastiana and Karydia.

**Figure 10:**
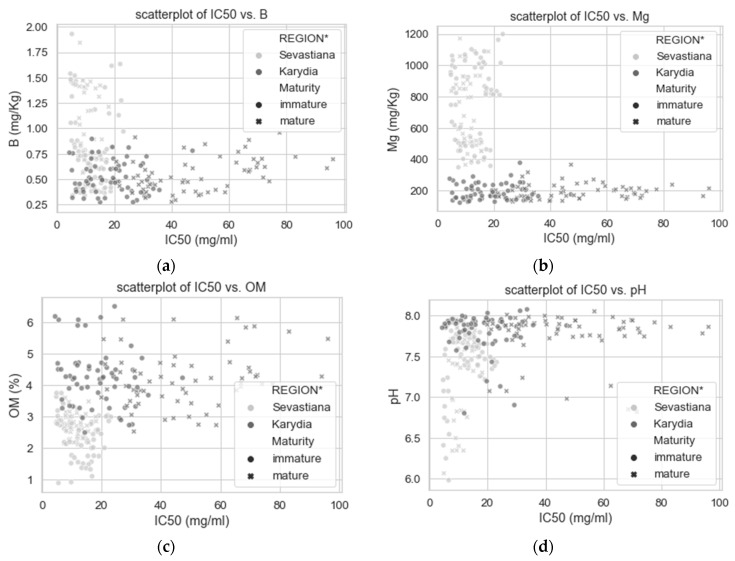

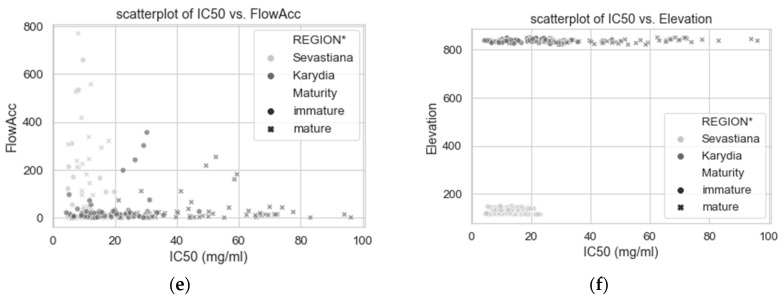
Scatter plots spotting data from Sevastiana area having low IC_50_ values in the DPPH assay at (**a**) specific levels of boron (B), (**b**) magnesium (Mg), (**c**) organic matter content (OM), (**d**) soil acidity (pH), (**e**) flow accumulation (FlowAcc) and (**f**) elevation.

The two experimental areas had significantly different climatic conditions, as it is evident by the mean temperature of May (Figure 9). Figure 10 shows the effect of some of the soil properties on the antioxidant capacity of cherry fruits. For example, all cherry trees grown on soils having boron concentration above 1 mg kg^−1^ produced fruits with IC50 values in the DPPH assay lower than 20 mg/mL (Figure 10a). Cherry trees also grown on soils having magnesium levels above 400 mg kg^−1^ (considered above optimum, Heckman [69]) produced fruits with IC50 values in the DPPH assay lower than 20 mg/mL (Figure 10b). Moreover, Figure 10c shows that most of the cherry trees grown in soils having less than 3% OM had IC50 values in the DPPH assay lower than 20 mg/mL, thus higher antioxidant activity. Highly acidic soils tend to have low DPPH values (Figure 10d). Increased flow accumulation (FlowAcc), which is a measure of the surface water drainage, promoted the antioxidant capacity of cherry fruits (Figure 10e). Finally, almost all the fruits from Sevastiana region located at 100 m above sea level had IC50 values in the DPPH assay lower than 20 mg/mland there were not mature fruits from the Karydia region located at about 900 m above the sea level having IC50 values in the DPPH assay lower than 20 mg/mL, as shown in Figure 10f.

Multicollinearity diagnostics (Mctest) was conducted for detecting collinearity in the dataset among regressors on the DPPH variable. The variance inflation factor (VIF) was computed, and variables with VIF values above 10 were removed (data not presented here). The variables removed for collinearity were Organic Matter, Phosphorus, pH, Sand content, Silt content, minimum temperature, wind speed and the latitude and longitude coordinates. Despite that XGBoost and Random forests are relative insensitive to multicollinearity, the performance of these models was better after the removal of the redundant regressors, and thus the same variables were used for all the prediction algorithms during their configuration.

**Figure 11:**
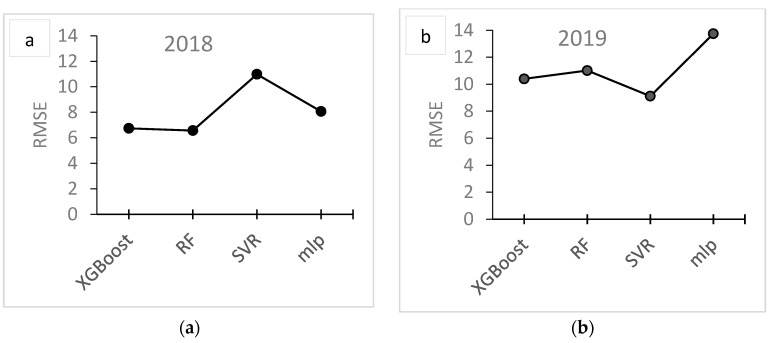

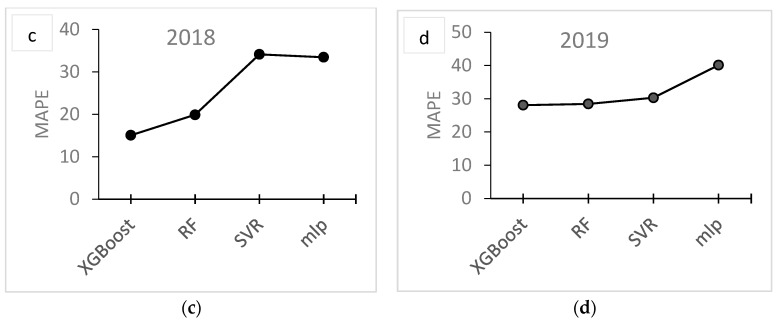
Root mean square error (RMSE) (**a**,**b**) and mean absolute percentage error (**c**,**d**) of DPPH prediction using XGBoost, random forest (RF), support vector regression (SVR) and multiple perceptron (MLP) models for 2018 (**a**–**c**) and 2019 (**b**,**d**).

Figure 11 shows the performance of the models used for predicting DPPH. For 2018, the smaller RMSE and MAPE achieved were 6.74 and 15.06, respectively, using the XGBoost algorithm. For 2019, using the algorithms trained on the 2018 data, the smaller RMSE achieved was 9.12 using the SVR algorithm, and the smaller MAPE achieved was 28.07 using the XGBoost algorithm. As the MAPE accuracy measurement is less sensitive to the outliers, which is quite common for agricultural modelling data, as opposed to the RMSE, the XGBoost is finally selected as the best predictive algorithm for DPPH.

**Figure 12:**
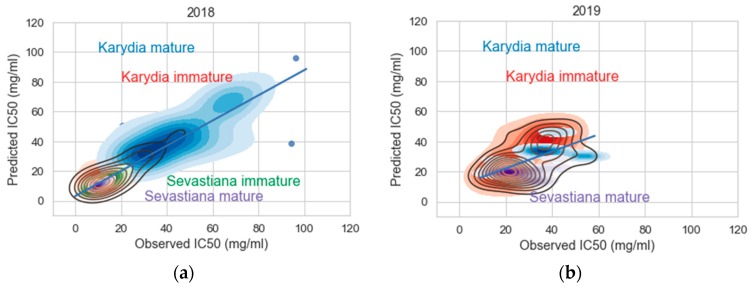
Kernel density plots showing the relationship between observed and predicted IC50 values in the DPPH assay using the XGBoost model for (**a**) 2018 and (**b**) 2019.

Figure 12 shows the relationship between the observed and predicted IC50 values in the DPPH assay for 2018 and 2019 using the XGBoost model. The kernel density plots show that for both 2018 and 2019, there are two central regions for Sevastiana and Karydia mature fruits, respectively, containing the highest density of DPPH values for these fruit classes, and they are separable on the x- and y-axes both for 2018 and 2019 cropping seasons. The IC50 values in the DPPH assay for Sevastiana mature fruits of the central region in 2018 were close to 10 mg/mL, whereas they were close to 20 mg/mL in 2019, meaning that 2018 was a better cropping year in terms of antioxidant activity for Sevastiana area. Unlike to Sevastiana, the IC50 values for the DPPH assay in the central density region were close to 30 mg/mL for both 2018 and 2019. Figure 12 indicates that the predictive model can identify the different classes of antioxidant activity of cherry fruits.

## 4. Discussion

The objective of this study was to investigate the efficacy of machine learning model to predict the antioxidant capacity of cherry fruits using remote sensing, soil analysis, climatic, topographic and hydrographic data. The results of the predictions indicate the feasibility of characterizing the free radical scavenging activity (DPPH) using remote sensing indices, soil attributes, climatic, topographic and hydrographic data. However, further investigation is required, as just one year was used for validation and the antioxidant activity for Sevastiana in 2019 was relatively lower compared to 2018 impairing a little the correlation between the observed and the predicted values. However, the results of the present study are promising, as this work is the first attempt to use remote sensing, soil analysis, climatic, topographic and hydrographic data for the prediction of the antioxidant capacity of cherry fruits.

The chemical composition of the fruits (i.e., the FCR reducing substances) was evaluated in order to associate the antioxidant capacity of the samples to the other measured parameters. The weak correlation between DPPH and FCR in 2018 (Figure 3) can be attributed to the increased FCR concentration of the immature cherry fruits harvested from Karydia compared to the cherry fruits harvested from Sevastiana in 2018 (Figure 4). As immature fruits were not harvested from Sevastiana region in 2019, the correlation between DPPH and FCR was found to be strong. Results shown in Figure 4 show that immature fruits harvested from Sevastiana had lower FCR and DPPH levels compared to immature fruits harvested from Karydia, which was not an expected finding, as it is known that phenolic compounds have antioxidant activity [70]. Zargoosh et al. [71] observed the same positive correlation between DPPH and phenolic content for one out of the three regions, where they conducted their experiments for figwort (*Scrophularia striata*) antioxidant potential. They attributed this positive correlation to the formation of other compounds that affect the antioxidant potential of plants; however, further research is necessary for the identification of these compounds.

Anthocyanins, a group of flavonoids, have two absorption maxima of the solar radiation, one between 270 and 290 nm and the other in the visible spectrum at 500–550 nm (Woodall and Stewart, 1998). Interestingly, Table 4 shows that DPPH was mostly correlated with the Carotenoid Reflectance Index 2 (CRI2) from the first UAS flight compared to the other indices and the CRI2 index was a highly contributing variable in the PC1 (Figure 2). As the CRI2 index uses a band centred at 510 nm (close to the blue region of the spectrum), it probably captured the effect of flavonoids on the antioxidant capacity of cherry fruits before maturity. Thus, the higher antioxidant capacity of immature fruits harvested from Sevastiana could probably be attributed to the higher flavonoid content of cherry trees grown in Sevastiana compared to Karydia. However, the establishment of a relationship between the antioxidant activity and a specific plant compound is difficult due to the complexity of the compounds in plants. The absorption of anthocyanins peaks occurred at ~550 nm according to Gitelson et al. [45]. This is probably why the importance of the ARI index, which uses the 550 nm band, is evaluated to be higher compared to the CRI2 index from the XGBoost algrorithm both for the DPPH and FCR (Figure 5). The difference between PCA and XGBoost in the estimation of the importance of features is that the XGBoost estimates the feature importance from a trained predictive model and it is a supervised machine learning technique (the dataset has target variable).

The higher FCR of Karydia immature harvested fruits can be attributed to the lower temperatures recorded for Karydia compared to Sevastiana. Figure 6 shows that for May the mean temperature was 27.7% higher for Sevastiana compared to Karydia. Temperature influences the development of phenolic compounds both at high and low temperatures (Sun et al. 2017). A reduction in these compounds at high temperatures can be due to temperature induced transcriptome changes regulating the biosynthetic pathway of phenolic compounds [72]. The mature fruits in Karydia were harvested in June, when probably the temperature effect was eliminated and thus mature fruits from Karydia and Sevastiana did not have significantly different FCR (Figure 4).

Despite the FCR being higher for immature cherry fruits harvested from Karydia compared to immature fruits harvested from Sevastiana for 2018, the antioxidant activity was higher for Sevastiana compared to Karydia for both mature and immature fruits (Figure 7a,b). Figure 7 shows that EC and DPPH have high contribution on PC1 and opposite directions on axis 1. Thus, the higher antioxidant activity of fruits harvested from Sevastiana can be attributed to the soil attributes. Many studies have shown that increased EC causes metabolic processes that increase phenolic, flavonoid and oxidative enzymes, which in turn increase the antioxidant capacity of plant organs [73,74,75]. This is because phenolics, including flavonoids, are important for the plant defence system against environmental stress, such as high EC [76]. Extremely high magnesium concentrations in soil, along with other soil attributes such as high boron, manganese concentrations, etc., caused the production of fruit with high antioxidant properties, which is evident in Figure 10. Moreover, highly acidic soils tend to have low DPPH values, probably because of higher boron availability of these soils (Figure 10b). It is known that boron soil fixation is increased at pH levels above 7 [77]. Increased flow accumulation (FlowAcc), which is a measure of the surface water drainage, promoted the antioxidant capacity of cherry fruits (Figure 10e). This is an interesting finding because, apart from the group of cherry trees grown on soils with high EC that promoted high antioxidant capacity of their fruits, there is another group of trees grown on sandy soils, with poor nutrient availability or leached due to high surface water drainage that also exhibit high antioxidant potential. This is also confirmed by Figure 4, which shows that soil sand levels (S) and DPPH have high contribution on PC1 and opposite directions on axis 1. In general, the soil properties (mainly electrical conductivity, iron, manganese and boron concentrations, magnesium and potassium concentrations, and organic matter content) are more important parameters for the free radical scavenging activity (DPPH) of cherries compared to elevation. However, elevation is more important parameter for FCR, especially at the plant growth stage before fruit maturation.

The most interesting correlations for leaf analysis and antioxidant capacity of cherry fruits was the negative correlation of leaf boron content (L_B) with DPPH, validating the importance of soil boron on the antioxidant capacity of cherry fruits, and the correlation of leaf potassium content (L_K) with the FCR (Table 4). The latter agrees with Oliveira et al. [23], who showed that potassium fertilization in greater amounts than optimum for maximum growth and yield, promoted the development of phenolic compounds in plants. Potassium levels were above the optimum for obtaining the maximum yield for almost all cherry trees in the two experimental areas (data not presented here).

## 5. Conclusions

The basic hypothesis of this research, that remote sensing data in combination with soil data are necessary and efficient in predicting antioxidant content in cherries in near real-time and certainly in advance of harvesting, was confirmed. Specifically, the research findings revealed the potential to support delineation of uniform land units with the best response to the maximum antioxidant capacity of cherry fruits towards site-specific harvesting (precision farming), with an analogous marketing perspective.

Within the context of this study, an algorithm based on machine learning gradient boosting decision tree was produced for predicting the antioxidant activity of cherries by means of remote sensing. This methodology could be expanded to mapping and assessment of the total cherry production area of an area for the antioxidant activity of cherries aiming at disposing at market fruits labeled as having various levels of antioxidants for targeting public groups having high requirements of high antioxidant capacity fruits (anti-inflammatory, cardioprotective, cancer chemopreventive and neuroprotective requirements).

This study also showed that the soil properties and elevation had a significant role on the antioxidant activity and FCR of cherries, respectively. Interestingly, although the experimental area, located at 100 m above the sea level (Sevastiana), produced fruits with higher antioxidant capacity for both mature and immature fruits, the immature fruits produced at 900 m above the sea level (Karydia) had higher FCR, possibly because of the lower air temperature at plant growth stage before fruit maturation. Thus, the soil properties (mainly, electrical conductivity, iron, manganese and boron concentrations, magnesium and potassium concentrations and organic matter content) are more important parameters for the free radical scavenging activity (DPPH) compared to elevation. Unlike to DPPH, the FCR is mostly affected by the elevation.

## Figures and Tables

**Table 1:** Overall figures of experimental data collection.

Year	Site	Fields	Area (ha)	Fruit Samples	Soil Samples	Images ^1^
2018	Sevastiana	4	1.54	108	31	5
	Karydia	8	1.87	123	33	4
	Total	12	3.41	231	64	9
2019	Sevastiana	4	1.54	18	10	2
	Karydia	8	1.87	31	9	3
	Total	12	3.41	49	19	5

^1^ The number of images is equivalent to the dates of sampling.

**Table 2:** The sites, the cherry varieties and the dates of sample collection for 2018.

Sites	Variety	Date of Sample Collection								
		28/4	4/5	9/5	16/5	23/5	25/5	29/5	5/6	13/6
Sevastiana	Lapins	√	√	√	√	√				
	Sabrina		√	√	√					
	Early Lory	√	√							
	Canada Giant	√	√	√	√	√				
	Sweet Early	√	√	√						
Karydia	Ferrovia						√	√	√	√
	Hedelfinger						√	√	√	√
	Germersdorfer						√	√	√	√
	Bakirtzeika						√	√	√	√

**Table 3:** The sites, the cherry varieties and the dates of fruit sample collection for 2019.

Sites	Variety	Date of Sample Collection					
		10/5	21/5	9/5	30/5	7/6	21/6
Sevastiana	Lapins	√	√	√			
	Sabrina		√	√			
	Canada Giant	√	√	√			
Karydia	Ferrovia				√	√	√
	Hedelfinger				√	√	√
	Germersdorfer				√	√	√
	Bakirtzeika				√	√	√

**Table 4:** Pearson’s r correlation between soil, remote sensing and climatic attributes with DPPH and FCR.

Variable	Correlation for DPPH		Correlation for FCR	
	Pearson’s r	*p*-Value	Pearson’s r	*p*-Value
FCR	−0.400	0.000 *		
BRIX	0.116	0.080	−0.493	0.000 *
VARIETY	−0.162	0.014 *	−0.162	0.014 *
DATE	0.678	0.000 *	−0.081	0.223
B	−0.253	0.000 *	0.017	0.796
C	0.028	0.676	0.024	0.721
CaCO_3_	0.214	0.001 *	0.115	0.082
Cu	0.241	0.000 *	0.138	0.035 *
EC	−0.369	0.000 *	−0.124	0.060
Fe	0.221	0.000 *	0.097	0.142
K	0.017	0.798	0.008	0.899
Mg	−0.465	0.000 *	−0.058	0.381
Mn	−0.311	0.000 *	0.064	0.335
N	−0.126	0.055	−0.054	0.412
OM	0.42	0.000 *	0.173	0.009 *
P	0.128	0.050 *	0.092	0.161
pH	0.262	0.000 *	−0.049	0.455
S	−0.503	0.000 *	−0.161	0.015 *
Si	0.545	0.000 *	0.165	0.012 *
Zn	0.069	0.29	0.058	0.384
FlowAcc	−0.125	0.058	−0.032	0.632
Aspect	0.071	0.286	−0.016	0.812
Elevation	0.568	0.000 *	0.042	0.027 *
Slope	0.303	0.000 *	−0.170	0.529
m_temp	−0.210	0.000 *	−0.167	0.010 *
h_temp	−0.293	0.000 *	−0.049	0.011*
l_temp	−0.157	0.017 *	−0.133	0.462
rain	0.038	0.569	−0.133	0.045 *
wind_sp	−0.564	0.000 *	−0.042	0.523
wind_dir	−0.289	0.000 *	−0.164	0.013 *
ARI_1	0.208	0.002 *	−0.042	0.523
ARI_2	−0.224	0.000 *	−0.124	0.060
CRI2_1	0.451	0.000 *	0.086	0.192
CRI2_2	−0.045	0.495	0.065	0.329
NDVI_1	0.311	0.000 *	0.032	0.630
NDVI_2	0.039	0.551	0.085	0.198
L_N	−0.325	0.020	−0.195	0.169
L_P	−0.007	0.961	−0.234	0.098
L_K	−0.189	0.184	0.416	0.002 *
L_Ca	0.043	0.765	0.234	0.098
L_Mg	−0.050	0.716	−0.246	0.081
L_B	−0.416	0.002	0.052	0.716
L_Mn	−0.222	0.117	−0.170	0.233
L_Zn	−0.212	0.117	−0.356	0.010 *
L_Fe	−0.211	0.137	−0.438	0.001 *
L_Cu	0.074	0.605	−0.407	0.003 *

* Significance ≤ 0.05. FCR: Folin−Ciocalteu reducing capacity; B: boron; C: clay; CaCO_3_: calcium carbonate; Cu: copper; EC: electrical conductivity; Fe: iron; K: potassium; Mg: magnesium; Mn: manganese; N: nitrate/nitrogen; OM: organic matter; P: phosphorus; S: sand; Si: silt; Zn: zinc; FlowAcc: flow accumulation; m_temp: minimum temperature; h_temp: high temperature; l_temp: low temperature; wind_sp: wind speed; wind_dir: wind direction; L_N: leaf nitrogen; L_P: leaf phosphorus; L_K: leaf potassium; L_Ca: leaf calcium; L_Mg: leaf magnesium; L_B: leaf boron; L_Mn: leaf managanese; L_Zn: leaf zinc; L_Fe: leaf iron; L_Cu: leaf copper.
